# Association between antibiotics use and diabetes incidence in a nationally representative retrospective cohort among Koreans

**DOI:** 10.1038/s41598-021-01125-5

**Published:** 2021-11-04

**Authors:** Sun Jae Park, Young Jun Park, Jooyoung Chang, Seulggie Choi, Gyeongsil Lee, Joung Sik Son, Kyae Hyung Kim, Yun Hwan Oh, Sang Min Park

**Affiliations:** 1 Department of Biomedical Sciences, Seoul National University Graduate School, Seoul, 03082 Korea; 2 Department of Family Medicine, Seoul National University Hospital, Seoul National University College of Medicine, 101, Daehak-ro, Jongno-gu, Seoul, 03080 Korea; 3 Department of Family Medicine, Korea University Guro Hospital, Seoul, 08308 Korea; 4 Department of Family Medicine, Jeju National University Hospital, Jeju National University College of Medicine, Jeju, 63241 Korea

**Keywords:** Diabetes, Diabetes

## Abstract

Numerous studies have reported that antibiotics could lead to diabetes, even after adjusting for confounding variables. This study aimed to determine the causal relationship between antibiotics use and diabetes in a nationally representative cohort. This retrospective cohort study included adults aged 40 years or older who were enrolled in the Korean National Health Insurance Service-Health Screening Cohort. Antibiotic exposure was assessed from 2002 to 2005 and newly diagnosed diabetes mellitus was determined based on diagnostic codes and history of antidiabetic medication use from 2006 to 2015. Multivariate Cox proportional hazards model was used to assess the association between antibiotic use and diabetes incidence. The mean age of the 201,459 study subjects was 53.2 years. People who used antibiotics for 90 or more days had a higher risk of diabetes (adjusted hazard ratio [aHR] 1.16, 95% confidence interval [CI] 1.07–1.26) compared to non-users. Those who used five or more classes of antibiotics had a higher risk of diabetes than those who used one antibiotic class (aHR 1.14; 95% CI 1.06–1.23). The clear dose-dependent association between antibiotics and diabetes incidence supports the judicious use of antibiotics in the future.

## Introduction

Diabetes mellitus (DM) is a chronic metabolic disease characterized by elevated fasting serum glucose concentration, impaired glucose tolerance, and elevated glycated hemoglobin level^1^. DM is often accompanied by dyslipidemia^2^, hypertension, or obesity^3^ and is a criteria of metabolic syndrome. DM affects approximately 400 million people worldwide. The prevalence of DM has quadrupled over the past three decades^4^. Many nations have prioritized the prevention of DM and careful treatment of already affected patients during this worldwide pandemic. Although various causes such as genetic predisposition, unhealthy diet, and sedentary lifestyle are important drivers of DM^5^, the pathophysiology and etiology of this disease remain unelucidated.

The gut microbiota comprises microorganisms that exist symbiotically but can be easily perturbed by medications^6,7^. Previous studies have explored the effect of antibiotics on gut microbiota in animal models and in human prospective cohorts^8,9^. Broad-spectrum antibiotics affect the gut community, causing rapid declines in taxonomic richness and diversity^10^. In infants and children, with undeveloped microbiomes, the effect of antibiotics on the gut microbiome may be larger. Observational studies have shown that early antibiotic use could lead to obesity, which could be mediated by changes in the gut microbiome^11–13^.

The close relationship between obesity and DM has been reported. Excess adiposity is the strongest risk factor for type 2 and non-insulin-dependent DM^3^. Along with obesity, the effects of antibiotics on the microbiome that could lead to type 2 DM has been investigated^14–16^. However, the association between antibiotic use and DM incidence in these studies is inconsistent^17^ and studies on this relationship are lacking in Asian populations. Moreover, the results of previous studies have been partly confounded by body mass index (BMI), lifestyle factors, household income, and family history of DM^16^. In addition, previous studies were prone to recall bias regarding antibiotic use^17^. Thus, a large population cohort study including healthy adults to determine the relationship between antibiotic use and DM is warranted. Therefore, we analyzed data from the Korean National Health Insurance Service-Health Screening Cohort (NHIS-HEALS) database^18,19^, a nationally representative cohort among Koreans, to assess the association between antibiotic use and DM incidence.

## Methods

### Data source and study design

This study used data from the NHIS-HEALS (NHIS-2020-2-089) 2002–2015. This database is a cohort of participants who underwent health screening provided by the NHIS. In South Korea, insurants aged ≥ 40 years are eligible for general national health screening biennially. From this health screening examination, the database contains information on demographic variables, health behaviors, laboratory results, records of inpatient and outpatient clinic use, and prescription records. The national healthcare claims database managed by the government, such as NHIS-HEALS, covers approximately 98% of the population of South Korea and can be used for research. The NHIS provides its database for research purposes, such as epidemiological studies, and its validity has been described in detail elsewhere^20,21^. This population-based cohort study was approved by the Institutional Review Board of Seoul National University Hospital (IRB number: E-2104-195-1214). The requirement for informed consent from the participants was waived by the Institutional Review Board of Seoul National University Hospital as the NHIS-HEALS database is anonymized according to strict confidentiality guidelines. This study adhered to the principles of the Declaration of Helsinki and all methods were carried out in accordance with relevant guidelines and regulations.

### Study population

**Figure 1:**
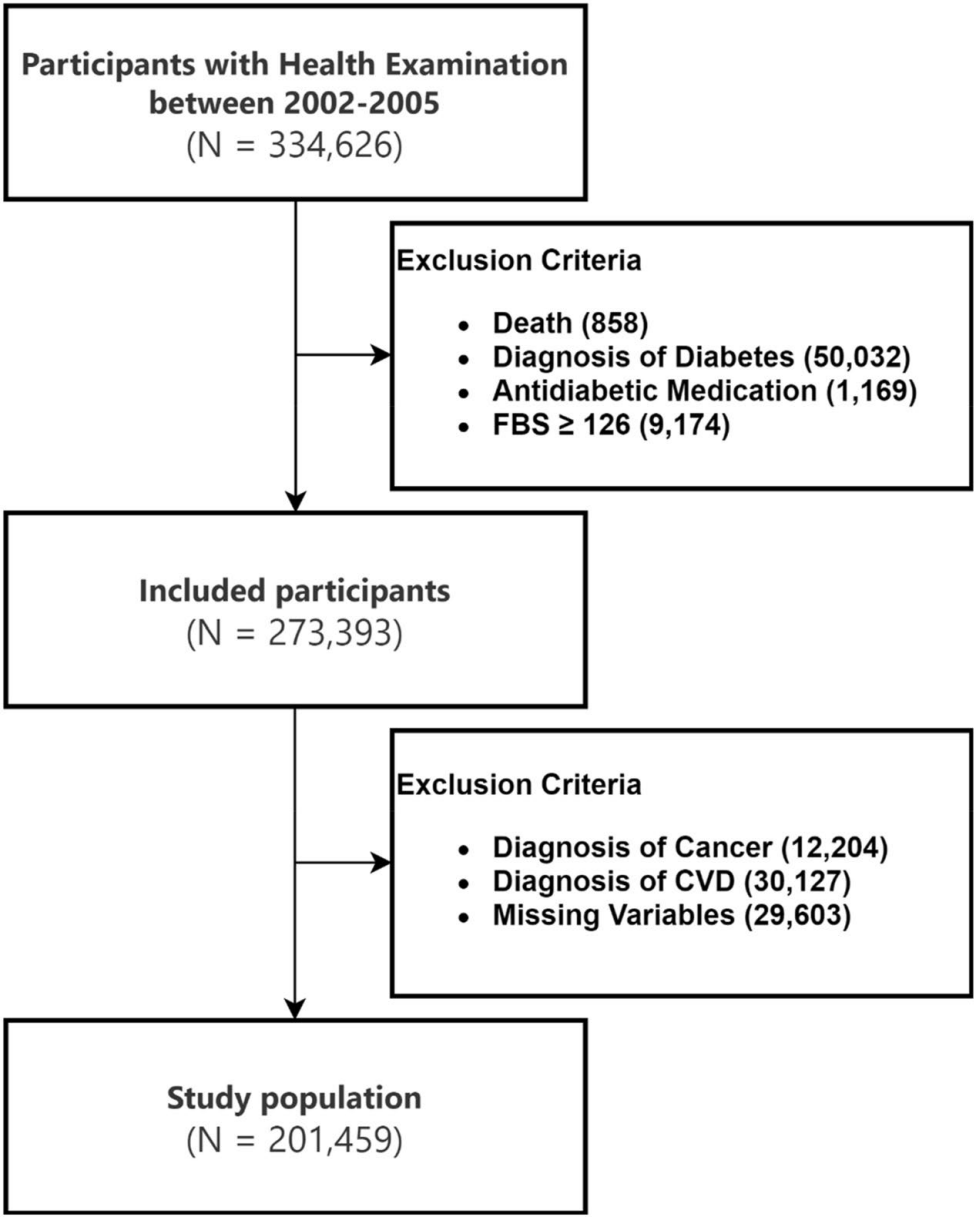
Study sample selection.

The NHIS-HEALS database provides health screening data for individuals aged 40–79 years in 2002^19^. We identified 334,626 participants who underwent health screening in 2002–2005 and excluded 858 participants who died before the index date of January 1, 2006, 50,032 participants diagnosed with DM before the index date, 1169 participants who had already been prescribed antidiabetic medication, 9174 participants with fasting blood sugar (FBS) levels ≥ 126 mg/dL, 12,204 participants diagnosed with cancer, and 30,127 participants diagnosed with cardiovascular disease (CVD) before the index date to correct for other possible reasons for antibiotic use such as underlying infection in immunocompromised patients. Moreover, we excluded participants with missing variables. Finally, the analysis included data from 201,459 participants (Fig. 1).

### Key variables

#### Main outcome variable

The main outcome was a new diagnosis of DM between 2006 and 2015. DM incidence was identified as newly appearing International Classification of Diseases, 10th revision (ICD-10) codes (E10-E14) and the prescription of antidiabetic medication (Supplementary Table 1). Person-years and adjusted hazard ratios (HRs) were calculated based on the first date of DM diagnosis.

#### Ascertainment of antibiotic use

The exposure variables were the numbers of cumulative days of antibiotic prescription and antibiotic classes from 2002 to 2005. Antibiotic classes were determined using the claim database and defined according to the World Health Organization (WHO) Anatomical Therapeutic Chemical (ATC) classification of drugs as macrolides, penicillin, cephalosporin, fluoroquinolones, sulfonamides, lincosamides, tetracyclines, and others^22–24^. (Supplementary Table 2) The number of cumulative days of antibiotic prescription was categorized into 0, 1–29, 30–89, and 90 or more days. The number of antibiotic classes was categorized as 0, 1, 2, 3, 4, and 5 or more. The cumulative days of antibiotics use and the number of antibiotic classes were extracted separately in the NHIS database. Statistical analyses were done independently in separate models thereby removing the possibility of interaction between these two variables. The antibiotic non-user or the lowest antibiotic user groups were used as the reference groups for analyses^12^. When adjusting for the indications for antibiotic use, namely, the infection sources, the lowest antibiotic user group was used as the reference to avoid multicollinearity. The application of operational definitions of antibiotics use of this study and previous studies have been provided in Supplementary Table 1.

#### Ascertainment of covariates

The considered covariates included age (continuous, years), sex (categorical, men and women), BMI (categorical, < 18.5, 18.5–22.9, 23–24.9, and ≥ 25 kg/m^2^), smoking (categorical, never smoker, past smoker, and current smoker), alcohol consumption (categorical, none, ≤ 2, and ≥ 3 times weekly), physical activity (categorical, none, 1–4, and 5–7 times per week), household income (categorical, first, second, third, and fourth quartiles), residence (categorical, capital, metropolitan, and rural), family history of DM (categorical, yes and no), Charlson comorbidity index (CCI, categorical, 0, 1–2, and ≥ 3), FBS (continuous, mg/dL), total cholesterol (continuous, mg/dL), acid suppressants use (categorical, yes and no), and infectious diseases (categorical, yes and no). Infectious diseases include respiratory diseases; urinary tract infections; skin, soft tissue, bone, and joint infections (SSTBJ); and intra-abdominal infections (Supplementary Table 3).

BMI was calculated by dividing the weight in kilograms by the squared value of height in meters and was categorized as underweight, normal, overweight, or obese (< 18.5, 18.5–22.9, 23–24.9, and ≥ 25 kg/m^2^, respectively) based on the Asian Pacific criteria of the WHO^25^. Household income was derived from the insurance premiums. The first and fourth quartiles represented the lowest and highest household income, respectively. The CCI, which includes comorbidities such as chronic obstructive pulmonary disease and asthma, was used to consider comorbidities from claims data^26^. The use of acid suppressants, defined as histamine-2-receptor antagonists and proton pump inhibitors, were also recorded in the claims database.

The reasons for antibiotic use; in other words, the indications for antibiotic use, were considered as covariates. Five systems were considered: respiratory diseases, urinary tract infections, SSTBJ, intra-abdominal infections, and others (Supplementary Table 3)^27–29^. The major and most widely prevalent infection diagnoses were considered for each system. These sources of infections were considered to account for major confounding factors as DM can trigger infection and lead to the use of antibiotics. When ascertaining the diagnosis of infectious diseases, the NHIS database searched within one main diagnosis that the physician allocated.

### Statistical analysis

Multivariate Cox proportional hazards models were used to estimate the adjusted hazard ratios (aHRs) and 95% confidence intervals (CIs) of DM according to antibiotic use after adjusting for all the covariates described above. For analyses, antibiotic non-users were the reference group to assess the risk of the cumulative days of antibiotic prescription. Furthermore, the group that used only one class of antibiotics was set as a reference for the analysis of antibiotic class number, as the fully adjusted models considered the source of infection as a covariate. In addition, washout periods of 1, 2, and 3 years were applied to the subjects to minimize protopathic bias^30^. Furthermore, we conducted stratified subgroup analyses for all covariates. All data mining and analyses were performed using SAS version 9.4 (SAS Institute, Cary, NC, USA). Statistical significance was defined as a *P* < 0.05.

## Results

The baseline characteristics of the 201,459 participants are presented in Table 1. The antibiotic non-user group comprised 24,178 individuals. Among antibiotic users, the subgroups of cumulative days of antibiotic prescription (1–29, 30–89, and ≥ 90 days) contained 107,618, 55,958, and 13,705 participants, respectively. The mean (standard deviation) age of the total population was 53.18 (8.5) years. Compared to antibiotic non-users, participants with longer antibiotic use (≥ 90 days) tended to have higher BMI and more often used acid suppressants. The descriptive characteristics of participants diagnosed with infectious diseases are described in Supplementary Table 4. Although the patients with antibiotics prescriptions were 177,281, the number of infectious disease diagnoses was less than 64,807, which is further discussed afterward.

**Table 1:** Cohort characteristics.

	Total population	Antibiotics non-user	Antibiotics User		
			Number of cumulative days prescribed		
			1–29	30–89	≥ 90
**Number of people**	201,459	24,178	107,618	55,958	13,705
**Age, years, mean (SD)**	53.18 (8.5)	52.64 (8.1)	52.83 (8.3)	53.67 (8.7)	54.91 (9.2)
**Sex, N (%)**					
Men	115,517 (57.34)	17,343 (71.73)	63,529 (59.03)	27,674 (49.45)	6971 (50.86)
Women	85,942 (42.66)	6835 (28.27)	44,089 (40.97)	28,284 (50.55)	6734 (49.14)
**Body Mass Index, kg/m**^**2**^**, N (%)**					
< 18.5	4649 (2.31)	674 (2.79)	2478 (2.30)	1180 (2.11)	317 (2.31)
18.5 ≤ BMI < 23	77,024 (38.23)	9544 (39.47)	41,462 (38.53)	21,057 (37.63)	4961 (36.20)
23 ≤ BMI < 25	56,515 (28.05)	6662 (27.55)	30,150 (28.02)	15,847 (28.32)	3856 (28.14)
25 ≤ BMI	63,271 (31.41)	7298 (30.18)	33,528 (31.15)	17,874 (31.94)	4571 (33.35)
**Smoking, N (%)**					
Never smoker	137,521 (68.26)	14,063 (58.16)	72,046 (66.95)	41,245 (73.71)	10,167 (74.18)
Past smoker	19,385 (9.62)	2546 (10.53)	10,614 (9.86)	4948 (8.84)	1277 (9.32)
Current smoker	44,553 (22.12)	7569 (31.31)	24,958 (23.19)	9765 (17.45)	2261 (16.50)
**Alcohol consumption, times per week, N (%)**					
None	111,480 (55.34)	11,486 (47.51)	57,859 (53.76)	33,433 (59.75)	8702 (63.50)
1–2	69,207 (34.35)	9548 (39.49)	38,118 (35.42)	17,537 (31.34)	4004 (29.22)
≥ 3	20,772 (10.31)	3144 (13.00)	11,641 (10.82)	4988 (8.91)	999 (7.29)
**Physical activity, times per week, N (%)**					
None	100,112 (49.69)	11,720 (48.47)	53,077 (49.32)	28,313 (50.60)	7002 (51.09)
1–4	81,888 (40.65)	10,280 (42.52)	44,362 (41.22)	21,996 (39.31)	5250 (38.31)
5–7	19,459 (9.66)	2178 (9.01)	10,179 (9.46)	5649 (10.10)	1453 (10.60)
**Household income, N (%)**					
First quartile (lowest)	27,665 (13.73)	3413 (14.12)	14,555 (13.52)	7730 (13.81)	1967 (14.35)
Second quartile	42,251 (20.97)	4983 (20.61)	22,531 (20.94)	11,831 (21.14)	2906 (21.20)
Third quartile	57,089 (28.34)	6624 (27.40)	30,570 (28.41)	15,949 (28.50)	3946 (28.79)
Fourth quartile (highest)	74,454 (36.96)	9158 (37.88)	39,962 (37.13)	20,448 (36.54)	4886 (35.65)
**Residence, N (%)**					
Capital	78,555 (38.99)	10,500 (43.43)	42,162 (39.18)	20,959 (37.45)	4934 (36.00)
Metropolitan	47,951 (23.80)	5478 (22.66)	25,644 (23.83)	13,545 (24.21)	3284 (23.96)
Rural	74,953 (37.21)	8200 (33.92)	39,812 (36.99)	21,454 (38.34)	5487 (40.04)
**Family history of diabetes, N (%)**					
No	190,138 (94.38)	22,855 (94.53)	101,585 (94.39)	52,722 (94.22)	12,976 (94.68)
Yes	11,321 (5.62)	1323 (5.47)	6033 (5.61)	3236 (5.78)	729 (5.32)
**Charlson Comorbidity Index, N (%)**					
0	94,149 (46.73)	18,841 (77.93)	56,012 (52.05)	16,599 (29.66)	2697 (19.68)
1–2	100,325 (49.80)	5206 (21.53)	49,334 (45.84)	36,150 (64.60)	9635 (70.30)
3 or more	6985 (3.47)	131 (0.54)	2272 (2.11)	3209 (5.73)	1373 (10.02)
**Fasting blood sugar, mean (SD)**	91.32 (12.1)	92.02 (12.4)	91.37 (12.1)	91.00 (12.0)	90.98 (12.1)
**Total cholesterol, mean (SD)**	197.99 (36.0)	197.46 (36.4)	197.88 (35.8)	198.29 (36.3)	198.52 (36.3)
**Acid suppressants use, N (%)**					
No	55,784 (27.69)	15,353 (63.50)	32,820 (30.50)	6584 (11.77)	1027 (7.49)
Yes	145,675 (72.31)	8825 (36.50)	74,798 (69.50)	49,374 (88.23)	12,678 (92.51)

*SD* standard deviation, *N* number of people.

The association between the cumulative days of antibiotic prescription and DM incidence are presented in Table 2 and Supplementary Table 5. We observed a clear dose–response relationship between DM and cumulative antibiotic prescription days. A fully adjusted Cox proportional hazards model (Model 3) showed a higher risk of DM in the group with ≥ 90 days antibiotic use (aHR 1.16, 95% CI 1.07–1.26) compared to non-users. Furthermore, all models showed a higher risk with the long-term use (≥ 90 days). In Model 3, the aHRs for DM risk in the 30–89 and 1–29-day antibiotic use groups were 1.04 (95% CI 0.97–1.11) and 0.98 (95% CI 0.93–1.04) compared to non-users (p for trend < 0.001). Using the 1–29 cumulative days of as the reference, the dose–response relationship was unchanged (p for trend < 0.001) and ≥ 90 days of antibiotic use remained statistically significant (aHR 1.18, 95% CI 1.11–1.26). We also observed a dose–response relationship between DM and cumulative antibiotic days in a fully adjusted Cox proportional hazards model including infectious diseases (Model 3) and a higher risk of DM for ≥ 90 days of antibiotic use (aHR 1.19, 95% CI 1.11–1.27) compared to 1–29 days of antibiotic use (Supplementary Table 5).

**Table 2:** Hazard ratios for diabetes by number of cumulative days antibiotics prescribed.

	Antibiotics non-user	Number of cumulative days antibiotics prescribed			*p* for trend
		1–29	30–89	≥ 90	
**Events, N**	1586	7047	3989	1144	
**Person-years, 10**^**4**^	23	103	53	13	
**aHR (95% CI)**					
Model 1	1.00 (ref.)	1.00 (0.94 1.05)	1.07 (1.01 1.13)	1.20 (1.11 1.30)	< 0.001
Model 2	1.00 (ref.)	1.01 (0.96 1.07)	1.09 (1.03 1.16)	1.24 (1.15 1.34)	< 0.001
Model 3	1.00 (ref.)	0.98 (0.93 1.04)	1.04 (0.97 1.11)	1.16 (1.07 1.26)	< 0.001
**aHR (95% CI)**					
Model 1	1.01 (0.95 1.06)	1.00 (ref.)	1.07 (1.03 1.11)	1.21 (1.13 1.29)	< 0.001
Model 2	0.99 (0.94 1.05)	1.00 (ref.)	1.08 (1.04 1.13)	1.23 (1.16 1.31)	< 0.001
Model 3	1.02 (0.96 1.08)	1.00 (ref.)	1.06 (1.02 1.10)	1.18 (1.11 1.26)	< 0.001

*aHR* adjusted hazard ratio, *CI* confidence interval, *ref.* reference.

Model 1 Adjusted for age, sex, and body mass index.

Model 2 Adjusted for Model 1 plus smoking status, days with alcohol drinking per week, physical activity, household income, and residence.

Model 3 Adjusted for Model 2 plus family history of diabetes, Charlson comorbidity index, fasting blood sugar, total cholesterol, and acid suppressants use.

The results of the subgroup analyses stratified by all covariates in Model 3 are presented in Table 3. A total of 13,766 patients were newly diagnosed DM during the follow-up period. We also conducted analyses with washout periods. When 1, 2, and 3-year washout period were included, compared to the antibiotic non-user group, the aHRs for ≥ 90 days of antibiotic use were 1.13 (95% CI 1.04–1.23), 1.12 (95% CI 1.03–1.22), and 1.11 (95% CI 1.01–1.22), respectively. The risk of DM was higher in the long-term antibiotic use group. The association between antibiotic use and the risk of DM incidence was unlikely to be modified by age, BMI, smoking status, alcohol consumption, physical activity, household income, family history of DM, CCI, total cholesterol, and acid suppressants use.

**Table 3:** Sensitivity analyses and stratified analyses of association between antibiotics use and diabetes incidence.

	Total	Event	aHR (95% CI) by the number of cumulative days antibiotics prescribed				
			Non-user	1–29	30–89	≥ 90	*p* for interaction
**Sensitivity analyses**							
Wash-out period							
No wash-out (Main)	201,459	13,766	1.00 (ref.)	0.98 (0.93 1.04)	1.04 (0.97 1.11)	1.16 (1.07 1.26)	
1-year wash-out	200,693	13,000	1.00 (ref.)	0.98 (0.93 1.04)	1.03 (0.97 1.10)	1.13 (1.04 1.23)	
2-year wash-out	199,722	12,029	1.00 (ref.)	0.98 (0.92 1.04)	1.03 (0.96 1.10)	1.12 (1.03 1.22)	
3-year wash-out	198,538	10,845	1.00 (ref.)	0.97 (0.91 1.03)	1.03 (0.96 1.10)	1.11 (1.01 1.22)	
**Stratified analyses**							
Age							
< 60 years	156,487	9,552	1.00 (ref.)	1.02 (0.95 1.09)	1.05 (0.98 1.13)	1.20 (1.09 1.33)	0.691
≥ 60 years	44,972	4,214	1.00 (ref.)	0.90 (0.81 1.01)	1.01 (0.89 1.13)	1.07 (0.93 1.24)	
Body Mass Index							
< 25 kg/m^2^	138,188	6,564	1.00 (ref.)	0.92 (0.85 1.00)	0.98 (0.90 1.08)	1.14 (1.02 1.28)	0.407
≥ 25 kg/m^2^	63,271	7,202	1.00 (ref.)	1.04 (0.96 1.13)	1.09 (0.99 1.19)	1.17 (1.04 1.31)	
Smoking status							
Never	137,521	8,598	1.00 (ref.)	0.98 (0.91 1.06)	1.07 (0.98 1.16)	1.13 (1.01 1.25)	0.623
Ever	63,938	5,168	1.00 (ref.)	0.97 (0.89 1.05)	0.97 (0.88 1.07)	1.22 (1.07 1.39)	
Alcohol consumption days/week							
Never	111,480	7,362	1.00 (ref.)	1.00 (0.92 1.09)	1.10 (1.00 1.20)	1.19 (1.06 1.33)	0.599
1 day or over	89,979	6,404	1.00 (ref.)	0.97 (0.90 1.05)	0.98 (0.90 1.07)	1.14 (1.01 1.29)	
Physical activity, times/week							
Never	100,112	7,095	1.00 (ref.)	1.01 (0.93 1.09)	1.06 (0.97 1.16)	1.24 (1.11 1.39)	0.939
1 time or over	101,347	6,671	1.00 (ref.)	0.96 (0.88 1.03)	1.01 (0.93 1.11)	1.07 (0.95 1.21)	
Household income							
Lower half	69,916	5,206	1.00 (ref.)	0.98 (0.89 1.07)	1.06 (0.95 1.17)	1.22 (1.07 1.39)	0.571
Upper half	131,543	8,560	1.00 (ref.)	0.99 (0.92 1.06)	1.03 (0.95 1.11)	1.12 (1.01 1.25)	
Family history of diabetes							
No	190,138	12,581	1.00 (ref.)	0.97 (0.92 1.03)	1.03 (0.96 1.10)	1.16 (1.06 1.26)	0.301
Yes	11,321	1,185	1.00 (ref.)	1.10 (0.90 1.33)	1.13 (0.91 1.41)	1.13 (0.84 1.52)	
Charlson Comorbidity Index							
0	94,149	5,853	1.00 (ref.)	1.02 (0.96 1.09)	1.03 (0.95 1.13)	1.06 (0.90 1.25)	0.491
1 or ever	107,310	7,913	1.00 (ref.)	0.90 (0.81 1.00)	0.97 (0.87 1.08)	1.08 (0.96 1.22)	
Total cholesterol							
< 200 mg/dL	109,864	6,080	1.00 (ref.)	0.99 (0.91 1.08)	1.05 (0.96 1.16)	1.12 (0.99 1.27)	0.374
≥ 200 mg/dL	91,595	7,686	1.00 (ref.)	0.98 (0.90 1.05)	1.03 (0.95 1.12)	1.19 (1.07 1.33)	
Acid suppressants use							
No	55,784	3,459	1.00 (ref.)	1.02 (0.94 1.10)	1.08 (0.96 1.22)	1.00 (0.78 1.29)	0.288
Yes	145,675	10,307	1.00 (ref.)	0.94 (0.87 1.02)	1.00 (0.92 1.09)	1.13 (1.02 1.25)	

*aHR* adjusted hazard ratio, *CI* confidence interval, *ref.* reference.

Model Adjusted for age, sex, body mass index, smoking status, days with alcohol drinking per week, physical activity, household income, residence, family history of diabetes, Charlson comorbidity index, fasting blood sugar, total cholesterol, and acid suppressants use. The estimates were based on fully adjusted models.

The associations between DM incidence and number of antibiotic classes are shown in Table 4 and Supplementary Table 6. Compared to those who used one class of antibiotics, participants who used five or more antibiotic classes had a higher risk of DM (aHR 1.14, 95% CI 1.06–1.23), with indications for antibiotics included as covariates (Table 4). Representative examples of antibiotics for each antibiotic class and the major indications for antibiotic use are presented in Supplementary Tables 2 and 3. The analyses of antibiotic class number and DM incidence without indications for antibiotics as covariates are presented in Supplementary Table 6. Compared to the antibiotic non-user group, participants who used five or more antibiotic classes had a higher risk of DM incidence (aHR 1.11, 95% CI 1.03–1.19).

**Table 4:** Adjusted hazard of diabetes incidence according to the number of antibiotic classes among antibiotics users.

	Antibiotics class number					*p* for trend
	1	2	3	4	5 or more	
N of people (%)	34,686 (19.57)	44,631 (25.18)	44,708 (25.22)	33,275 (18.77)	19,981 (11.27)	
Events, N	2,247	2,930	3,098	2,401	1,504	
Person-years, 10^4^	33	43	43	32	19	
aHR (95% CI)	1.00 (ref.)	1.00 (0.95 1.06)	1.05 (0.99 1.11)	**1.08 (1.02 1.15)**	**1.14 (1.06 1.23)**	< 0.001

*N* number, *aHR* adjusted hazard ratio, *CI* confidence interval, *ref.* reference.

Model Adjusted for age, sex, body mass index, smoking status, days with alcohol drinking per week, physical activity, household income, residence, family history of diabetes, Charlson comorbidity index, fasting blood sugar, total cholesterol, acid suppressants use, and infectious diseases (respiratory diseases, urinary tract infections, skin, soft tissue, bone and joint infections, intra-abdominal infections, and others). The estimates were based on fully adjusted models.

Antibiotics were divided into seven classes consisting of penicillin, cephalosporin, macrolide, fluoroquinolone, sulfonamides, tetracyclines, and lincosamides or others.

Bold text means statistically significant hazards ratios, where the 95% confidence intervals do not include 1.

## Discussion

The results of this study provided real-world evidence of a dose-dependent relationship between DM incidence and the cumulative dose and number of classes of antibiotic prescriptions. This retrospective cohort study is the first in Asia to show this association between antibiotic use and newly diagnosed DM in a nationally representative population. Furthermore, we adjusted for various confounding factors, such as the indication for antibiotic use, and provided a wash-out analysis to address potential indication and protopathic biases.

Our results agree with those of previous studies showing an association between antibiotic use and DM incidence. Yuan et al.^17^ performed a prospective cohort study comprising 114,210 female US nurses with follow-up performed from 2008 to 2017. However, that study was limited by the inclusion of only women, the lack of analysis of individual antibiotic classes, and the risk for recall bias. A retrospective cohort study based on US veterans in New York by Davis et al.^15^ with follow-up performed from 2004 to 2014 showed that cephalosporin, macrolide, and penicillin use was associated with a higher incidence of DM. A Danish population-based case–control study by Mikkelsen et al.^16^ also suggested that antibiotic use could lead to DM; they also performed analysis for each antibiotic class.

Despite studies reporting a positive association, other studies have reported a null association between antibiotic use and DM. After rigorous adjustment for confounding factors such as BMI, ethnicity, location of residence, parental history of DM, diet, physical activity, and CCI that other studies could not take into consideration, a longitudinal cohort study in Alberta, Canada, observed no association between antibiotics and DM incidence^31^. Ye and others reported that previous studies based on administrative health databases were limited in controlling for important confounders regarding lifestyle behavior and socioeconomic and demographic factors. Nevertheless, while our study also adjusted for major confounding factors, we observed a positive relationship between antibiotic use and DM in a nationally representative cohort. Previous studies have reported a higher prevalence of type 2 DM in East Asians than in Europeans among people of similar BMI or waist circumference^32^. Thus, Spracklen et al.^33^ identified new genetic loci associated with type 2 DM in 433,540 East Asian individuals and performed the largest meta-analysis of type 2 DM in East Asian individuals. These cumulative findings support the need to study the association between antibiotic use and DM incidence, especially in East Asians or Koreans, where DM is more prevalent, partly due to genetic susceptibility.

**Figure 2:**
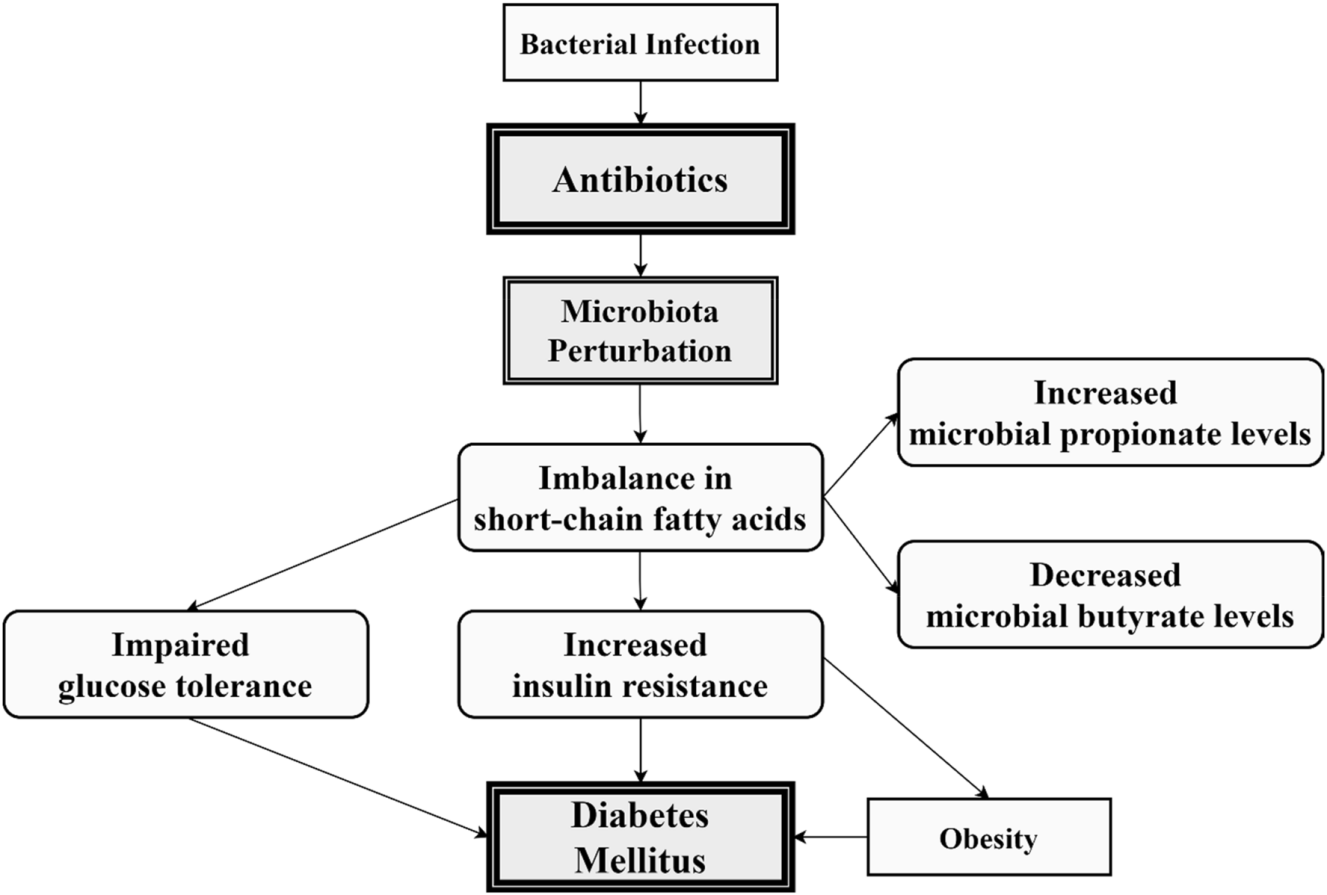
Potential hypotheses regarding relationship between antibiotics use and increased risk of diabetes.

Although several mechanisms for the relationship between antibiotics and DM incidence have been proposed, definitive evidence remains elusive. The present plausible mechanism indicated that antibiotics could perturb the gut microbiota, leading to DM in vulnerable populations. Data from animal models have shown that antibiotics can increase insulin resistance and alter the gut microbiota, increasing susceptibility to metabolic syndrome^34^. Individuals with pre-DM, type 2 DM, or metabolic syndrome have altered gut microbiota and an imbalance in the amount of short-chain fatty acids (SCFAs), which are intestinal metabolites that produce microbiota fermentation^35,36^. Other studies have shown that among SCFAs, increased butyrate levels led to improved insulin response in pancreatic beta-cells, while increased propionate caused by decreased host absorption increased the risk of type 2 DM^37^. The molecular mechanism and main hypotheses of the relationship between antibiotics and diabetes are illustrated in Fig. 2.

Our main results are presented with and without washout period analyses, both of which showed statistically significant associations between antibiotic use and DM incidence. Moreover, in the stratified analysis, no subgroup classification affected the association between antibiotics and DM. Age, BMI, lifestyle factors, household income, family history of DM, CCI, total cholesterol, and acid suppressant use did not influence the association between antibiotics and DM, even in the adjusted models. We included acid suppressant use as a covariate because proton pump inhibitors can also perturb the gut microbiota and contribute to the incidence of DM. Yuan et al.^38^ showed that the regular use of proton pump inhibitors was associated with a higher risk of type 2 DM and that the risk increased with longer duration of use based on the Nurses’ Health Study and Health Professionals Follow-up Study. In contrast, we did not observe an interaction between acid suppressant use and DM incidence, even in stratified analyses.

One issue worth addressing is the fact that although the number of antibiotics prescriptions was approximately 180,000, the number of infectious disease diagnoses was equal to or less than approximately one-third of this number. This particular phenomenon could have occurred because of two reasons. The first and main reason is that our analysis about the infectious diseases in the Korean NHIS-HEALS database only considered one main principal diagnosis for each patient’s hospital or clinic visit for analyzing accurate antibiotic indication information. Therefore, due to the characteristics and structures of the health claims data, the indications for using antibiotics could be contained in one of several subsidiary diagnoses that we could not detect. So, the underlying infections could not have been detected in our analysis. The second reason is the overprescription and widespread use of antibiotics in South Korean clinics compared to other Organization for Economic Co-operation and Development (OECD) countries as Park’s study pointed out^39^. Nevertheless, this serious overuse or misuse of antibiotics in Korea easily warranted and necessitated the need to evaluate and analyze the effect that antibiotics could have on other diseases, such as diabetes.

Robust sensitivity analyses and a clear dose-dependent relationship strengthened the credibility of our results. The strengths of our study also included its analysis of a large population from the NHIS comprising more than 200,000 individuals and the use of precise electronic medical records to avoid recall bias. In addition, the follow-up duration was > 10 years, which increased the credibility of our results. Moreover, we adjusted for potential confounding factors including age, BMI, lifestyle factors, household income, family history of DM, CCI, serum cholesterol levels, and acid suppressant use. We observed significantly higher HRs for DM incidence in participants who had used five or more cumulative antibiotic classes compared to those who used one class of antibiotics.

Our consideration of both the cumulative days of antibiotic prescription and number of antibiotic classes further strengthened our hypothesis. This study used separate Cox proportional hazards models regarding cumulative days of antibiotics subscriptions and the number of antibiotic classes subscriptions for the analyses. That is, the cumulative days of antibiotics and the number of antibiotics classes are independent variables in each different analysis. These various analyses were intended to evaluate the effect of antibiotic use on diabetes from various angles. Therefore, the possibility of an interaction between antibiotics class number and cumulative days used can be excluded. It is true that antibiotics class number and cumulative days prescribed may have a positive correlation. Those who used antibiotics longer could have tried many classes of antibiotics, possibly because of microorganisms resistant to antibiotics during the course of antibiotics prescription. However, these two proxies for the use of antibiotics were extracted by different methods from the NHIS database. In other words, two independent variables were considered for defining the use of antibiotics.

The fact that the indication for antibiotics was considered as a covariate also strengthens our results. The reason for this was that DM could be caused not only by antibiotics but also by the indication for the use of antibiotics. We also included washout periods of up to 3 years after antibiotic treatment to correct for protopathic biases caused by reverse causality. Finally, we excluded participants with pre-existing high fasting blood glucose levels to measure the incidence of newly occurring DM more accurately.

This study had several limitations. Although we excluded participants already prescribed antidiabetic medication or with high fasting blood glucose levels, there is the possibility of reverse causation due to undiagnosed prediabetic patients. DM can impair immune function; thus, diabetic patients are more prone to infections, which could increase their need for antibiotics. To overcome this shortcoming, our sensitivity analysis included wash-out period analyses, which revealed statistically significant HRs and also included the indication for antibiotics as covariates. Moreover, the NHIS-HEALS database did not provide the glycated hemoglobin values^19^ because this measurement was not included in routine check-ups. Also, our analysis containing infectious diseases as covariates included only one main diagnosis in the NHIS-HEALS database, as mentioned above, which can explain the discrepancy between those diagnosed with infectious diseases and the prescription of antibiotics. In other words, we included obvious indications for antibiotics in the analysis. In the future, studies that consider both main and several subsidiary diagnoses for antibiotic indications are needed. Furthermore, our study could not ascertain the exact DM type (type 1 or type 2), although a high percentage of newly diagnosed DM was presumed to be type 2^40^ considering the age of the total study population. Patients with type 1 DM are generally diagnosed earlier in life^41^. Finally, although our careful statistical analyses showed a dose–response relationship between antibiotics use and diabetes incidence, the fact that only 4 years of antibiotics use from 2002 to 2005 were considered is an inherent limitation of our study design. Nevertheless, newly diagnosed diabetes was ascertained from 2006 to 2015 and additional wash-out period analyses were performed to derive a causal relationship between antibiotics and diabetes. Further studies with longer and more timely follow-up periods are warranted in order to better explore this association. All in all, the significant dose–response relationship between antibiotics and DM incidence remained even after meticulous consideration of possible confounding variables. This finding suggests the need for the judicious prescription of antibiotics, especially in circumstances where antibiotic prescription is consistently increasing^42,43^.

Although these findings do not necessarily raise the need to change how we prescribe antibiotics or clinical practice guidelines at this current point, this retrospective cohort study does pose the possibility that antibiotics could raise the risk of diabetes in the future. Further human and animal studies are necessary to determine the exact causal relationship and mechanism of this phenomenon.

## Supplementary Information

Supplementary Information.

## Data Availability

To access the data from the NHIS database, researchers should be approved by the Korean National Health Insurance Service. After the approval, data are provided with anonymized personally identifiable information. Further information is available in online hompage (https://nhiss.nhis.or.kr).

## Abbreviations

aHR: Adjusted hazard ratio
ATC: Anatomical therapeutic chemical
BMI: Body mass index
CCI: Charlson comorbidity index
CI: Confidence interval
CVD: Cardiovascular disease
DM: Diabetes mellitus
FBS: Fasting blood sugar
HEALS: Health screening cohort
NHIS: National Health Insurance Service
ICD-10: International Classification of Diseases, 10th revision
SCFAs: Short chained fatty acids
SSTBJ: Skin, soft tissue, bone and joint infections
