# Supplementary material for: Association between antibiotics use and diabetes incidence in a nationally representative retrospective cohort among Koreans
Source: Sci Rep. 2021 Nov 4;11:21681. doi: 10.1038/s41598-021-01125-5 (PMC8568925; doi:10.1038/s41598-021-01125-5)
Supplement: Supplementary file 1 — Supplementary Information. [file 41598_2021_1125_MOESM1_ESM.docx]

**Association between antibiotics use and diabetes incidence in a nationally representative retrospective cohort among Koreans**

Sun Jae Park^1,†^, BSc, Young Jun Park^2,†^, MD, Jooyoung Chang^1^, MD, Seulggie Choi^1^, MD, Gyeongsil Lee^2^, MD, MSc, Joung Sik Son^3^, MD, PhD, Kyaehyung Kim^2^, MD, PhD, Yunhwan Oh^4^, MD, MSc, Sang Min Park^1,2,*^, MD, MPH, PhD

1. Department of Biomedical Sciences, Seoul National University Graduate School, Seoul 03082, Korea
2. Department of Family Medicine, Seoul National University Hospital, Seoul National University College of Medicine, Seoul 03080, Korea
3. Department of Family Medicine, Korea University Guro Hospital, Seoul 08308, Korea
4. Department of Family Medicine, Jeju National University Hospital, Jeju 63241, Korea

†These authors contributed equally to this work as co-first authors

**Supplementary information**

**Supplementary Table 1.** Comparative analysis of cohort studies dealing with antibiotics and diabetes incidence.

**Supplementary Table 2.** Examples of antibiotics for each class based on the World Health Organization Anatomical Therapeutic Chemical (WHO ATC) guidelines.

**Supplementary Table 3.** Representative sources of infection according to the ICD-10 codes, mainly categorized by systems.

**Supplementary Table 4.** Baseline characteristics for the indications for the use of antibiotics, classified by source of infection.

**Supplementary Table 5.** Hazard ratios for diabetes by number of cumulative days antibiotics prescribed in antibiotics users.

**Supplementary Table 6**. Adjusted hazard of diabetes incidence according to the number of antibiotic classes.

**Supplementary Table 1.** Comparative analysis of cohort studies dealing with antibiotics and diabetes incidence.

| Country | Database (Year) | Antibiotics (operational definitions) | Diabetes (operational definitions) | Study design | Reference |
| --- | --- | --- | --- | --- | --- |
| South Korea | the Korean National Health Insurance Service-Health Screening Cohort (NHIS-HEALS) (2002-2015) | Prescriptions in claim database defined according to the ATC classification of drugs (J01*) | ICD-10 codes (E10-E14) and the prescription of antidiabetic medications | Retrospective cohort study | This study |
| U.S. | the Nurses’ Health Study (NHS 2008–2014) and NHS II (2009–2017) | Questionnaires asking about total amount of time using antibiotics | Questionnaires asking whether participants had ever been diagnosed with diabetes | Prospective cohort study | Yuan et al. (2020)  (Reference #17) |
| U.S. | the Veterans Affairs New York Harbor Healthcare System (NYHHS) (2004-2014) | The number of courses of outpatient antibiotic prescriptions >6 months prior to date of diabetes diagnosis | Having ≥2 ICD-9 diagnosis codes (250, 357.2, 362.0, 366.4) from clinical encounters or ≥2 prescriptions of diabetes medications other than metformin | Retrospective cohort study | Davis et al. (2019)  (Reference #15) |
| Canada | Alberta’s Tomorrow  Project (ATP) – Alberta Health (AH) administrative datasets (2000-2015) | Antibiotic prescriptions according to ATC coding system (J01*) | Hospitalization with ICD code, physician claim diagnosis, using diabetes medication with ATC code for insulin (A10A) or glucose-lowering drugs (A10B) | Nested case-control study | Ye et al. (2018) (Reference #31) |
| Denmark | the Danish National Registry of Patients, the Danish National Prescription Registry, and the Danish Person Registry were combined (2000-2012) | Use of all systemic antibiotics (ATC, J01*, or P01AB01) from the cohort | A first-ever prescription of a noninsulin glucose-lowering agent (ATC A10B) | Case-control study | Mikkelsen et al. (2015)  (Reference #16) |

Acronyms; ATC, Anatomical Therapeutic Chemical

**Supplementary Table 2.** Examples of antibiotics for each class based on the World Health Organization Anatomical Therapeutic Chemical (WHO ATC) guidelines.

| **Antibiotics class** | **Types of antibiotics for each class** |
| --- | --- |
| Macrolides | erythromycin, spiramycin, midecamycin, oleandomycin, roxithromycin, josamycin, troleandomycin, clarithromycin, azithromycin, miocamycin, rokitamycin, dirithromycin, flurithromycin, telithromycin, solithromycin |
| Penicillins | ampicillin, pivamicillin, carbenicillin, amoxicillin, amoxicillin and clavulanate, azlocillin, mezlocillin, mecillinam, piperacillin, ticarcillin, metampicillin, talampicillin, dicloxacillin, oxacillin |
| Cephalosporins | cefalexin, cefaloridine, cefalotin, cefazolin, cefatrizine, ceftezole, cefoxitin, cefotetan, cefonicid, cefotaxime, ceftazidime, ceftriaxone, cefmenoxime, cefdinir, cefteram, cefepime, cefpirome, cefaclor, cefuroxime |
| Fluoroquinolones | ofloxacin, ciprofloxacin, pefloxacin, enoxacin, temafloxacin, norfloxacin, levofloxacin, moxifloxacin, gemifloxacin, gatifloxacin, sitafloxacin |
| Sulfonamides | sulfaisodimidine, sulfamethizole, sulfadimidine, sulfapyridine, sulfafurazole, sulfanilamide, sulfathiazole, sulfathiourea, sulfamethoxazole and trimethoprim, sulfadiazine, sulfasalazine, sulfamoxole |
| Lincosamides | clindamycin, lincomycin |
| Tetracyclines | doxycycline, tetracycline |

**Supplementary Table 3.** Representative sources of infection according to the ICD-10 codes, mainly categorized by systems.

| **Infectious diseases** | **ICD-10 codes** |
| --- | --- |
| **Respiratory diseases** |  |
| Pneumonia & Influenza | J09, J10, J11, J12, J13, J14, J15, J16, J17, J18 |
| Chronic bronchitis | J41, J42 |
| **Urinary tract infections (UTI)** |  |
| Cystitis | N30 |
| Acute pyelonephritis | N10 |
| Urethritis | N34, N37 |
| **Skin, soft tissue, bone and joint infections (SSTBJ)** |  |
| Cellulitis | L03 |
| Erysipelas | A46 |
| Impetigo | L01 |
| Folliculitis | L66.2, L66.4 |
| Furuncle & Carbuncle | L02 |
| Osteomyelitis | M86 |
| Synovitis | M65, M67, M68, M70 |
| **Intra-abdominal infections (IAI)** |  |
| Cholecystitis & Cholangitis | K80, K81, K83 |
| Appendicitis | K35, K36, K37 |
| Diverticulitis | K57 |
| Peritonitis | K65 |
| Pancreatitis | K85 |
| **Others** |  |
| Acute/chronic otitis media | H65, H66 |
| Sepsis | A40, A41 |
| Central nervous system infection | A81, A89 |

**Supplementary Table 4.** Baseline characteristics for the indications for the use of antibiotics, classified by source of infection.

|  | **Total population** | **Antibiotics non-user** | **Antibiotics User** | | |
| --- | --- | --- | --- | --- | --- |
|  |  |  | **Number of cumulative days prescribed** | | |
|  |  |  | **1-29** | **30-89** | **≥ 90** |
| **Number of people** | 201,459 | 24,178 | 107,618 | 55,958 | 13,705 |
|  |  |  |  |  |  |
| **Infectious Diseases** |  |  |  |  |  |
| **Respiratory diseases, N (%)** |  |  |  |  |  |
| No | 187,536 (93.09) | 24,178 (100) | 102,938 (95.65) | 49,535 (88.52) | 10,885 (79.42) |
| Yes | 13,923 (6.91) |  | 4,680 (4.35) | 6,423 (11.48) | 2,820 (20.58) |
| **Urinary tract infections (UTI), N (%)** |  |  |  |  |  |
| No | 181,579 (90.13) | 24,178 (100) | 100,869 (93.73) | 46,231 (82.62) | 10,301 (75.16) |
| Yes | 19,880 (9.87) |  | 6,749 (6.27) | 9,727 (17.38) | 3,404 (24.84) |
| **Skin, soft tissue, bone and joint infections (SSTBJ), N (%)** |  |  |  |  |  |
| No | 183,367 (91.02) | 24,178 (100) | 100,025 (92.94) | 47,970 (85.73) | 11,194 (81.68) |
| Yes | 18,092 (8.98) |  | 7,593 (7.06) | 7,988 (14.27) | 2,511 (18.32) |
| **Intra-abdominal infections (IAI), N (%)** |  |  |  |  |  |
| No | 199,662 (99.11) | 24,178 (100) | 106,856 (99.29) | 55,152 (98.56) | 13,476 (98.33) |
| Yes | 1,797 (0.89) |  | 762 (0.71) | 806 (1.44) | 229 (1.67) |
| **Others, N (%)** |  |  |  |  |  |
| No | 190,344 (94.48) | 24,178 (100) | 104,061 (96.69) | 50,755 (90.70) | 11,350 (82.82) |
| Yes | 11,115 (5.52) |  | 3,557 (3.31) | 5,203 (9.30) | 2,355 (17.18) |

Others include acute/chronic otitis media, sepsis and central nervous system infection.

**Supplementary Table 5.** Hazard ratios for diabetes by number of cumulative days antibiotics prescribed in antibiotics users.

|  | **Number of cumulative days antibiotics prescribed** | | |  |
| --- | --- | --- | --- | --- |
|  | **1-29** | **30-89** | **≥ 90** | ***p* for trend** |
| Events, N | 7,047 | 3,989 | 1,144 |  |
| Person-years, 10^4^ | 103 | 53 | 13 |  |
|  |  |  |  |  |
| aHR (95% CI) |  |  |  |  |
| Model 1 | **1.00 (ref.)** | 1.07 (1.03 1.11) | 1.21 (1.13 1.28) | <0.001 |
| Model 2 | **1.00 (ref.)** | 1.08 (1.04 1.13) | 1.23 (1.15 1.31) | <0.001 |
| Model 3 | **1.00 (ref.)** | 1.06 (1.02 1.11) | 1.19 (1.11 1.27) | <0.001 |

Acronyms; aHR, adjusted hazard ratio; CI, confidence interval; ref., reference.

Model 1 Adjusted for age, sex, and body mass index.

Model 2 Adjusted for Model 1 plus smoking status, days with alcohol drinking per week, physical activity, household income, and residence.

Model 3 Adjusted for Model 2 plus family history of diabetes, Charlson comorbidity index, fasting blood sugar, total cholesterol, acid suppressants use, and infectious diseases (respiratory diseases, urinary tract infections, skin, soft tissue, bone and joint infections, intra-abdominal infections, and others).

**Supplementary Table 6**. Adjusted hazard of diabetes incidence according to the number of antibiotic classes.

|  |  | **Antibiotics class number** | | | | |  |
| --- | --- | --- | --- | --- | --- | --- | --- |
|  | **Antibiotics non-user** | **1** | **2** | **3** | **4** | **5 or more** | ***p* for trend** |
| N of people (%) | 24,178 (12.00) | 34,686 (17.22) | 44,631 (22.15) | 44,708 (22.19) | 33,275 (16.52) | 19,981 (9.92) |  |
| Events, N | 1,586 | 2,247 | 2,930 | 3,098 | 2,401 | 1,504 |  |
| Person-years, 10^4^ | 23 | 33 | 43 | 43 | 32 | 19 |  |
|  |  |  |  |  |  |  |  |
| aHR (95% CI) | **1.00 (ref.)** | 0.97 (0.91 1.04) | 0.97 (0.92 1.04) | 1.02 (0.96 1.09) | 1.05 (0.98 1.12) | **1.11 (1.03 1.19)** | <0.001 |

Acronyms; N, number; aHR, adjusted hazard ratio; CI, confidence interval; ref., reference.

Model Adjusted for age, sex, body mass index, smoking status, days with alcohol drinking per week, physical activity, household income, residence, family history of diabetes, Charlson comorbidity index, fasting blood sugar, total cholesterol, and acid suppressants use. The estimates were based on fully adjusted models.

Antibiotics were divided into seven classes consisting of penicillin, cephalosporin, macrolide, fluoroquinolone, sulfonamides, tetracyclines, and lincosamides or others.
